# Supplementary material for: The N-terminal PA domains of signal-peptide-peptidase-like 2 (SPPL2) proteases impact on TNFα cleavage
Source: Commun Biol. 2025 Apr 30;8:686. doi: 10.1038/s42003-025-08102-y (PMC12043953; doi:10.1038/s42003-025-08102-y)

a

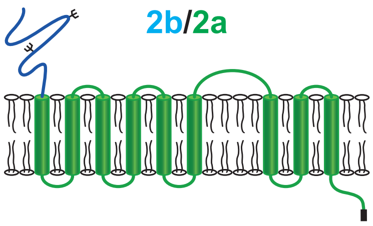


```
         10         20         30         40         50         60
MAAAVAAALA RLLAAFLLLA AQVACEYGMV HVVSQAGGPE GKDYCILYNP QWAHLPHDLS
         70         80         90        100        110        120
KASFLQLRNW TASLLCSAAD LPARGFSNQI PLVARGNCTF YEKVRLAQGS GARGLLIVSR
        130        140        150        160        170        180
ERLVPPGGNK TQYDEIGIPV ALLSYKDMLD IFTRFGRTVR AALYSPSWPN FDYTMVVIFV
        190        200        210        220        230        240
IAVFTVALGG YWSGLVELEN LKAVTTEDRE MRKKKEEYLT FSPLTVVIFV VICCVMMVLL
        250        260        270        280        290        300
YFFYKWLVYV MIAIFCIASA MSLYNCLAAL IHKIPYGQCT IACRGKNMEV RLIFLSGLCI
        310        320        330        340        350        360
AVAVVWAVFR NEDRWAWILQ DILGIAFCLN LIKTLKLPNF KSCVILLGLL LLYDVFFVFI
        370        380        390        400        410        420
TPFITKNGES IMVELAAGPF GNNEKLPVVI RVPKLIYFSV MSVCLMPVSI LGFGDIIVPG
        430        440        450        460        470        480
LLIAYCRRFD VQTGSSYIYY VSSTVAYAIG MILTFVVLVL MKKGQPALLY LVPCTLITAS
        490        500        510        520        530
VVAWRRKEMK KFWKGNSYQM MDHLDCATNE ENPVISGEQI VQQAYPYDVP DYA
```

b

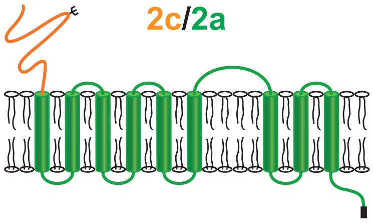


```
         10         20         30         40         50         60
MACLGFLLPV GFLLLISTVA GGKYGVAHVV SENWSKDYCI LFSSDYITLP RDLHHAPLLP
         70         80         90        100        110        120
LYDGTKAPWC PGEDSPHQAQ LRSPSQRPLR QTTAMVMRGN CSFHTKGWLA QGQGAHGLLI
        130        140        150        160        170        180
VSRVSDQQCS DTTLAPQDPR QPLADLTIPV AMLHYADMLD ILSHTRGEAV VRVAMYAPSW
        190        200        210        220        230        240
PNFDYTMVVI FVIAVFTVAL GGYWSGLVEL ENLKAVTTED REMRKKKEEY LTFSPLTVVI
        250        260        270        280        290        300
FVVICCVMMV LLYFFYKWLV YVMIAIFCIA SAMSLYNCLA ALIHKIPYGQ CTIACRGKNM
        310        320        330        340        350        360
EVRLIFLSGL CIAVAVVWAV FRNEDRWAWI LQDILGIAFC LNLIKTLKLP NFKSCVILLG
        370        380        390        400        410        420
LLLLYDVFFV FITPFITKNG ESIMVELAAG PFGNNEKLPV VIRVPKLIYF SVMSVCLMPV
        430        440        450        460        470        480
SILGFGDIIV PGLLIAYCRR FDVQTGSSYI YYVSSTVAYA IGMILTFVVL VLMKKGQPAL
        490        500        510        520        530        540
LYLVPCTLIT ASVVAWRRKE MKKFWKGNSY QMMDHLDCAT NEENPVISGE QIVQQAYPYD

VPDYA
```

c

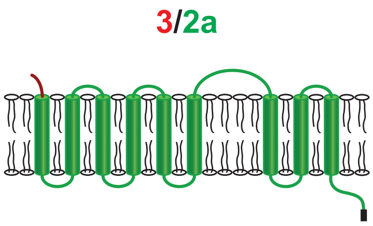


```
         10         20         30         40         50         60
MAEQTYSWAY SLVDYTMVVI FVIAVFTVAL GGYWSGLVEL ENLKAVTTED REMRKKKEEY
         70         80         90        100        110        120
LTFSPLTVVI FVVICCVMMV LLYFFYKWLV YVMIAIFCIA SAMSLYNCLA ALIHKIPYGQ
        130        140        150        160        170        180
CTIACRGKNM EVRLIFLSGL CIAVAVVWAV FRNEDRWAWI LQDILGIAFC LNLIKTLKLP
        190        200        210        220        230        240
NFKSCVILLG LLLLYDVFFV FITPFITKNG ESIMVELAAG PFGNNEKLPV VIRVPKLIYF
        250        260        270        280        290        300
SVMSVCLMPV SILGFGDIIV PGLLIAYCRR FDVQTGSSYI YYVSSTVAYA IGMILTFVVL
        310        320        330        340        350        360
VLMKKGQPAL LYLVPCTLIT ASVVAWRRKE MKKFWKGNSY QMMDHLDCAT NEENPVISGE
        370
QIVQQAYPYD VPDYA
```

Suppl. Figure 1: SPPL2a chimeric proteases. Schematic representation (left) and corresponding amino acid sequences in single letter code (right) of SPPL2b/2a (**a**), SPPL2c/2a (**b**) and SPPL3/2a (**c**). Protein domains of SPPL2a are depicted in green, those of SPPL2b in blue, SPPL2c in orange and SPPL3 in red. The HA-tag is illustrated as black box or in black letters, respectively. Bold letters indicate the amino acid shared in both proteases at the domain boarder. YD, GxGD, and PAL motifs are underlined.

a

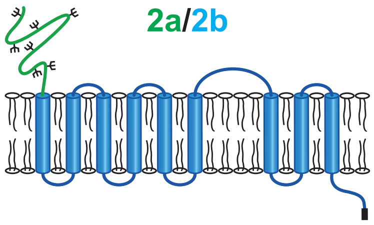


```
        10         20         30         40         50         60
MGPQRRLSPA GAALLWGFLL QLTAAQEAIL HASGNGTTKD YCMLYNPYWT ALPSTLENAT
        70         80         90        100        110        120
SISLMNLTST PLCNLSDIPP VGIKSKAVVV PWGSCHFLEK ARIAQKGGAE AMLVVNNSVL
       130        140        150        160        170        180
FPPSGNRSEF PDVKILIAFI SYKDFRDMNQ TLGDNITVKM YAPKEPVLDY NMVIIFIMAV
       190        200        210        220        230        240
GTVAIGGYWA GSRDVKKRYM KHKRDDGPEK QEDEAVDVTP VMTCVFVVMC CSMLVLLYYF
       250        260        270        280        290        300
YDLLVYVVIG IFCLASATGL YSCLAPCVRR LPFGKCRIPN NSLPYFHKRP QARMLLLALF
       310        320        330        340        350        360
CVAVSVVWGV FRNEDQWAWV LQDALGIAFC LYMLKTIRLP TFKACTLLLL VLFLYDIFFV
       370        380        390        400        410        420
FITPFLTKSG SSIMVEVATG PSDSATREKL PMVLKVPRLN SSPLALCDRP FSLLGFGDIL
       430        440        450        460        470        480
VPGLLVAYCH RFDIQVQSSR VYFVACTIAY GVGLLVTFVA LALMQRGQPA LLYLVPCTLV
       490        500        510        520        530        540
TSCAVALWRR ELGVFWTGSG FAKVLPPSPW APAPADGPQP PKDSATPLSP QPPSEEPATS
       550        560        570        580        590
PWPAEQSPKS RTSEEMGAGA PMREPGSPAE SEGRDQAQPS PVTQPGASAA YPYDVPDYA
```

b

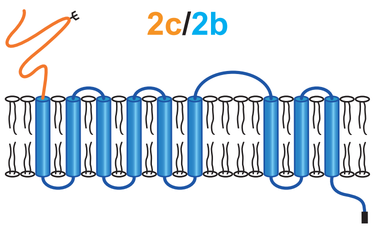


```
        10         20         30         40         50         60
MACLGFLLPV GFLLLISTVA GGKYGVAHVV SENWSKDYCI LFSSDYITLP RDLHHAPLLP
        70         80         90        100        110        120
LYDGTKAPWC PGEDSPHQAQ LRSPSQRPLR QTTAMVMRGN CSFHTKGWLA QGQGAHGLLI
       130        140        150        160        170        180
VSRVSDQQCS DTTLAPQDPR QPLADLTIPV AMLHYADMLD ILSHTRGEAV VRVAMYAPKE
       190        200        210        220        230        240
PVLDYNMVII FIMAVGTVAI GGYWAGSRDV KKRYMKHKRD DGPEKQEDEA VDVTPVMTCV
       250        260        270        280        290        300
FVVMCCSMLV LLYYFYDLLV YVVIGIFCLA SATGLYSCLA PCVRRLPFGK CRIPNNSLPY
       310        320        330        340        350        360
FHKRPQARML LLALFCVAVS VVWGVFRNED QWAWVLQDAL GIAFCLYMLK TIRLPTFKAC
       370        380        390        400        410        420
TLLLLVLFLY DIFFVFITPF LTKSGSSIMV EVATGPSDSA TREKLPMVLK VPRLNSSPLA
       430        440        450        460        470        480
LCDRPFSLLG FGDILVPGLL VAYCHRFDIQ VQSSRVYFVA CTIAYGVGLL VTFVALALMQ
       490        500        510        520        530        540
RGQPALLYLV PCTLVTSCAV ALWRRELGVF WTGSGFAKVL PPSPWAPAPA DGPQPPKDSA
       550        560        570        580        590        600
TPLSPQPPSE EPATSPWPAE QSPKSRTSEE MGAGAPMREP GSPAESEGRD QAQPSPVTQP
       610
GASAAYPYDV PDYA
```

c

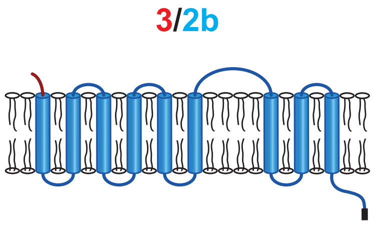


```
        10         20         30         40         50         60
MAEQTYSWAY SLVDYNMVII FIMAVGTVAI GGYWAGSRDV KKRYMKHKRD DGPEKQEDEA
        70         80         90        100        110        120
VDVTPVMTCV FVVMCCSMLV LLYYFYDLLV YVVIGIFCLA SATGLYSCLA PCVRRLPFGK
       130        140        150        160        170        180
CRIPNNSLPY FHKRPQARML LLALFCVAVS VVWGVFRNED QWAWVLQDAL GIAFCLYMLK
       190        200        210        220        230        240
TIRLPTFKAC TLLLLVLFLY DIFFVFITPF LTKSGSSIMV EVATGPSDSA TREKLPMVLK
       250        260        270        280        290        300
VPRLNSSPLA LCDRPFSLLG FGDILVPGLL VAYCHRFDIQ VQSSRVYFVA CTIAYGVGLL
       310        320        330        340        350        360
VTFVALALMQ RGQPALLYLV PCTLVTSCAV ALWRRELGVF WTGSGFAKVL PPSPWAPAPA
       370        380        390        400        410        420
DGPQPPKDSA TPLSPQPPSE EPATSPWPAE QSPKSRTSEE MGAGAPMREP GSPAESEGRD
       430        440
QAQPSPVTQP GASAAYPYDV PDYA
```

Suppl. Figure 2: SPPL2b chimeric proteases. Schematic representation (left) and corresponding amino acid sequences in single letter code (right) of SPPL2a/2b (**a**), SPPL2c/2b (**b**) and SPPL3/2b (**c**). Protein domains of SPPL2a are depicted in green, those of SPPL2b in blue, SPPL2c in orange and SPPL3 in red. The HA-tag is illustrated as black box or in black letters, respectively. Bold letters indicate the amino acid shared in both proteases at the domain boarder. YD, GxGD, and PAL motifs are underlined.

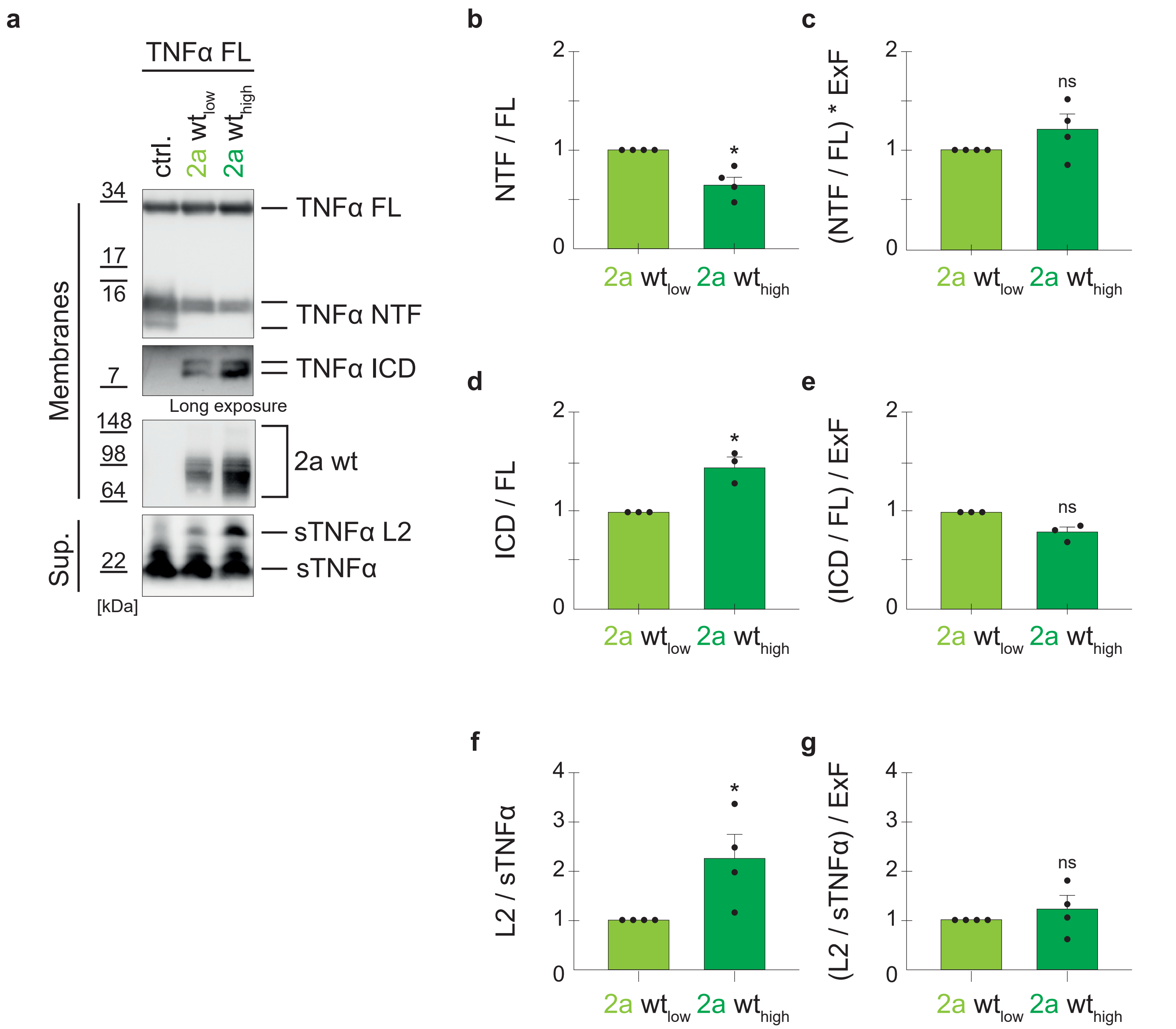

Suppl. Figure 3: Quantitative relationship between SPPL2a wt expression and TNFα processing. *Densitometric quantification confirms a linear dependency between TNFα NTF reduction, TNFα ICD production, sTNFα L2 production and SPPL2a wt expression.* (**a**) DKO cells (ctrl.) with either low (2a wt_{low}) or strong (2a wt_{high}) exogenous expression of SPPL2a wt were transiently transfected with full length TNFα (TNFα FL). Membrane bound TNFα species were analyzed on Western Blot using the anti-FlagM2 antibody. An antibody against the HA-tag (3F10) was used to detect SPPL2a wt expression. Soluble TNFα species were detected from Western Blot of conditioned media (sup.) with the monoclonal V5 antibody. (**b**) Densitometric quantification of TNFα NTF, as depicted in (a). Normalization to TNFα FL eliminated transfection variations. Resulting values were normalized to the low expressing 2a wt protease sample. *Note that high SPPL2a wt expression results in a significantly stronger reduction of TNFα NTF.* (**c**) Before normalization to SPPL2a wt_{low}, values as depicted in (b) were multiplied with an expression factor (ExF) accounting for the expression difference between high and low protease expression. *Note that this results in non-significant differences, indicating linearity between protease expression and TNFα NTF reduction.* (**d**) Densitometric quantification of TNFα ICD, as depicted in (a). Normalization to TNFα FL eliminated transfection variations. Resulting values were all normalized to the low expressing 2a wt protease sample. *Note that high SPPL2a wt expression results in a significantly stronger production of TNFα ICD.* (**e**) Before normalization to SPPL2a wt_{low}, values as depicted in (d) were divided by an expression factor (ExF) accounting for the expression difference between high and low protease expression. *Note that this results in non-significant differences, indicating linearity between protease expression and TNFα ICD production.* (**f**) Densitometric quantification of non-canonical TNFα shedding products (sTNFα L2) generated by SPPL2a wt as depicted in (a). Normalization to the ADAM-generated TNFα shedding product (sTNFα) eliminated transfection variations. Resulting values were all normalized to the low expressing 2a wt protease sample. *Note that high SPPL2a wt expression results in a significantly stronger secretion of sTNFα L2.* (**g**) Before normalization to SPPL2a wt_{low}, values as depicted in (f) were divided by an expression factor (ExF) accounting for expression difference between high and low protease expression. *Note that this results in non-significant differences, indicating linearity between protease expression and sTNFα secretion.* (**b**-**g**) Mean + SEM, unpaired, two-tailed one sample t-tests of log-transformed (log_2) values. ns=not significant, *$p < 0.05$ (b: p of 2a wt_{high}=0.0339; c: p of 2a wt_{high}=0.2682; d: p of 2a wt_{high}=0.0289; e: p of 2a wt_{high}=0.0637; f: p of 2a wt_{high}=0.0474; g: p of 2a wt_{high}=0.6339); b-c: n=4, D-E: n=3, F-G: n=4. The ExF is the mean (n=4) of the ratio between the expression of high and low expressing SPPL2a wt proteases.

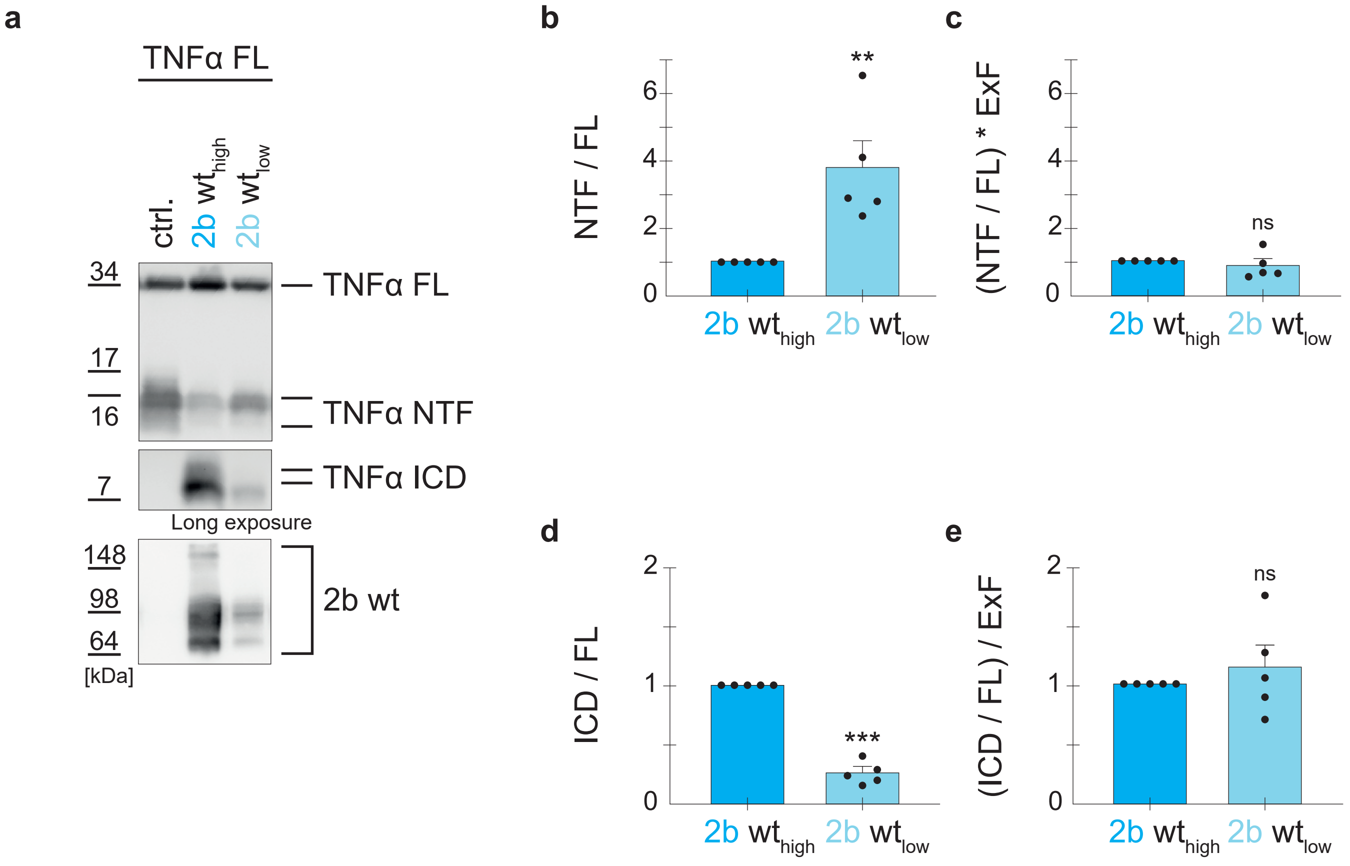

Suppl. Figure 4: Quantitative relationship between SPPL2b wt expression and TNFα processing. *Densitometric quantification confirms a linear dependency between TNFα NTF reduction, TNFα ICD production and SPPL2b wt expression.* (**a**-**e**) Experiments were carried out as described in Suppl. Figure 3, but using cells with different expression levels of SPPL2b wt and values were normalized to the high expressing SPPL2b wt sample. *Note that without consideration of the protease expression the low expressing SPPL2b wt cells show significantly reduced TNFα NTF turnover and TNFα ICD production. Significance is lost upon inclusion of ExF.* (**b**-**e**) Mean + SEM, unpaired, two-tailed one sample t-tests of log-transformed (log_2) values. ns=not significant, **p < 0.01 , ***p < 0.001 (b: p of 2b wt_{low}=0.0024; c: p of 2b wt_{low}=0.3006; d: p of 2b wt_{low}=0.0009; e: p of 2b wt_{low}=0.6182), n=5. The ExF is the mean (n=5) of the ratio between the expression of low and high expressing SPPL2b wt proteases.

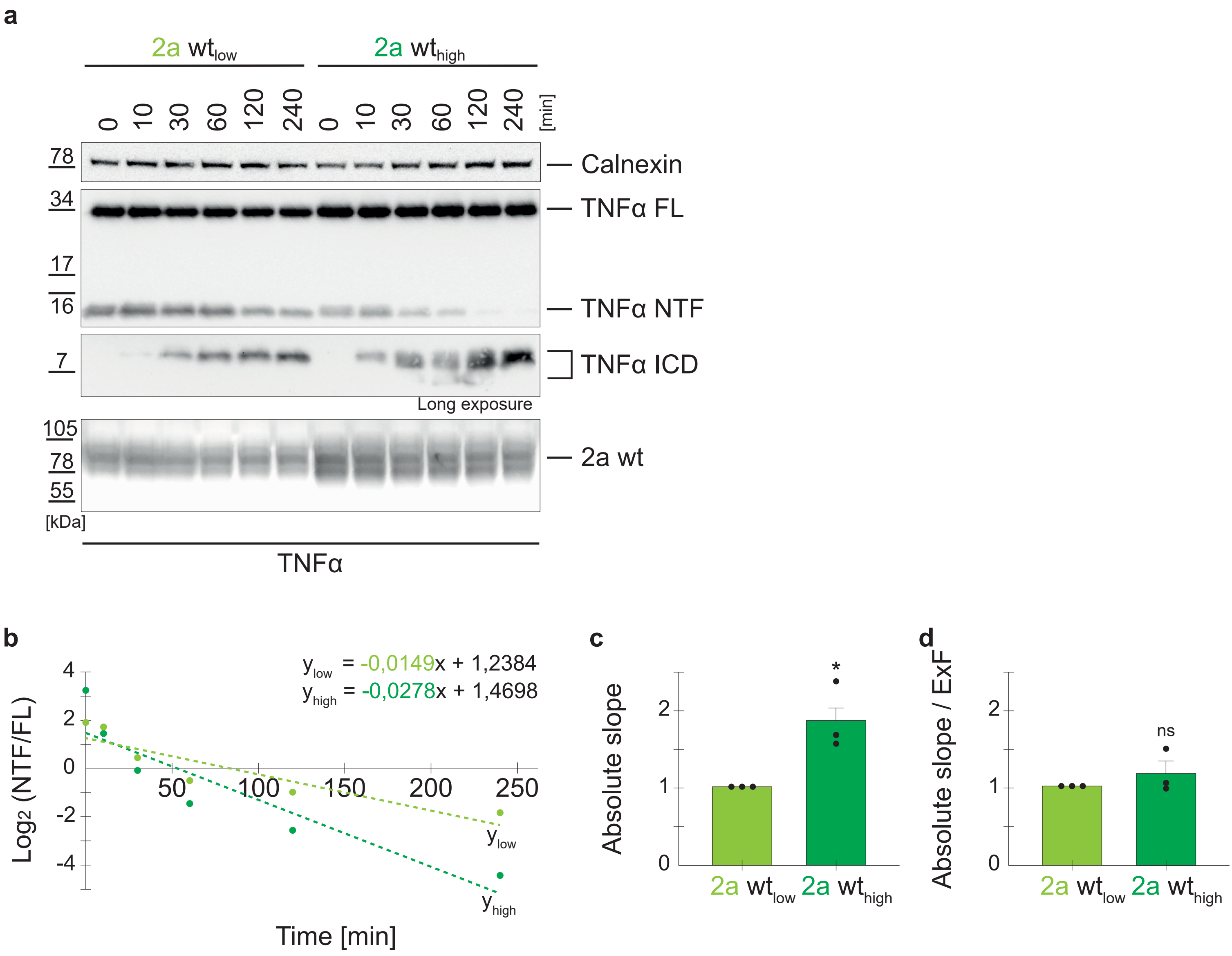

Suppl. Figure 5: Quantitative relationship between SPPL2a wt expression and kinetics of TNFα NTF turnover. *Densitometric quantification confirms a linear dependency between time dependent TNFα NTF reduction and SPPL2a wt protease expression level.* (**a**) DKO cells (crtl.) with either low (2a wt_{low}) or strong (2a wt_{high}) exogenous expression of SPPL2a wt were transiently transfected with full length TNFα (TNFα FL). Membrane fractions were incubated at 37 °C, and TNFα species were detected at the indicated time points on Western Blot using the Flag M2 antibody. An antibody against the HA-tag (3F10) was used to detect SPPL2a wt expression. Calnexin serves as a loading control. (**b**) Densitometric quantification of the TNFα NTF amount over time as depicted in (a). TNFα NTF at every time point was normalized to the respective calnexin value to eliminate variations in loading. The logarithmic values (log_2) of the result were plotted against time. The regression curves are displayed as dotted lines for y_{low} (SPPL2a wt_{low}) and y_{high} (SPPL2a wt_{high}). The corresponding slopes are marked in green. (**c**) The mean absolute slopes of 3 independent experiments as shown in (A) were depicted relative to the slope of samples from SPPL2a wt_{low}. *Note that high SPPL2a wt expression results in a significantly higher absolute slope.* (**d**) Values as depicted in (c) but expression differences between high and low expressing proteases are eliminated by division with the ExF before normalization to SPPL2a wt_{low}. *Note that this results in only non-significant differences, indicating linearity between protease expression and the slope, that reflects TNFα NTF reduction over time.* (**c&d**) Mean + SEM, unpaired, two-tailed one sample t-tests of log-transformed (log_2) values. ns= not significant, *$p < 0.05$ (c: p of 2a wt_{high}=0.0415; d: p of 2a wt_{high}=0.4037), n=3. The ExF is the mean (n=3) of the ratio between the expression of high and low expressing SPPL2a wt proteases.

a

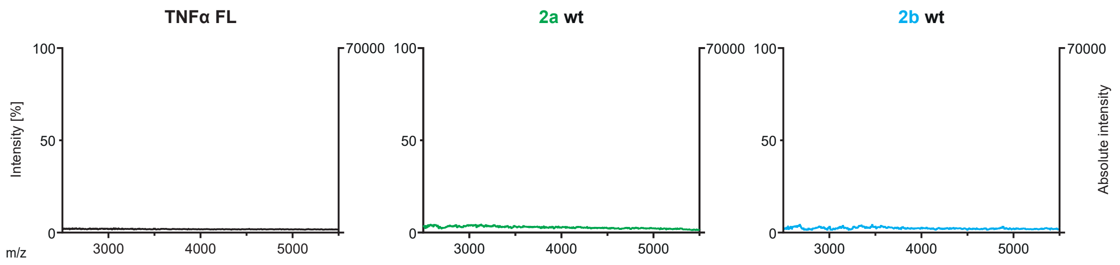

b

ICD (cleavage position)	Predicted mass [Da]	Measured mass [Da]			
		2a wt	2b/2a	2c/2a	3/2a
P18	3058	3065	3063	3062	3064
G26	3811	3816	3815	3814	3816
R28	4055	4054	4053	4052	4053
L39	5383	5383	5383	5383	5383
		2b wt	2a/2b	2c/2b	3/2b
P18	3058	3064	3066	3064	3065
G26	3811	3816	3819	3819	3816
R28	4055	4053	4054	4053	4054
L39	5383	5383	5383	5383	5383

Suppl. Figure 6: Empty controls and peak sizes, related to Figure 8. (**a**) Negative controls for mass spectrometry. DKO (ctrl.) cells were transiently transfected with full length TNFα (TNFα FL) or stably expressed either only SPPL2a wt (2a wt) or SPPL2b wt (2b wt). Mass spectrometric analysis of TNFα ICD species was carried out as in Fig. 8. No background peaks were detected. (**b**) Table of predicted and experimentally determined masses. Single letter code and numbers indicate position of the most N-terminal amino acid of the respective TNFα cleavage product.

Uncropped Western Blot images

Figure 2b

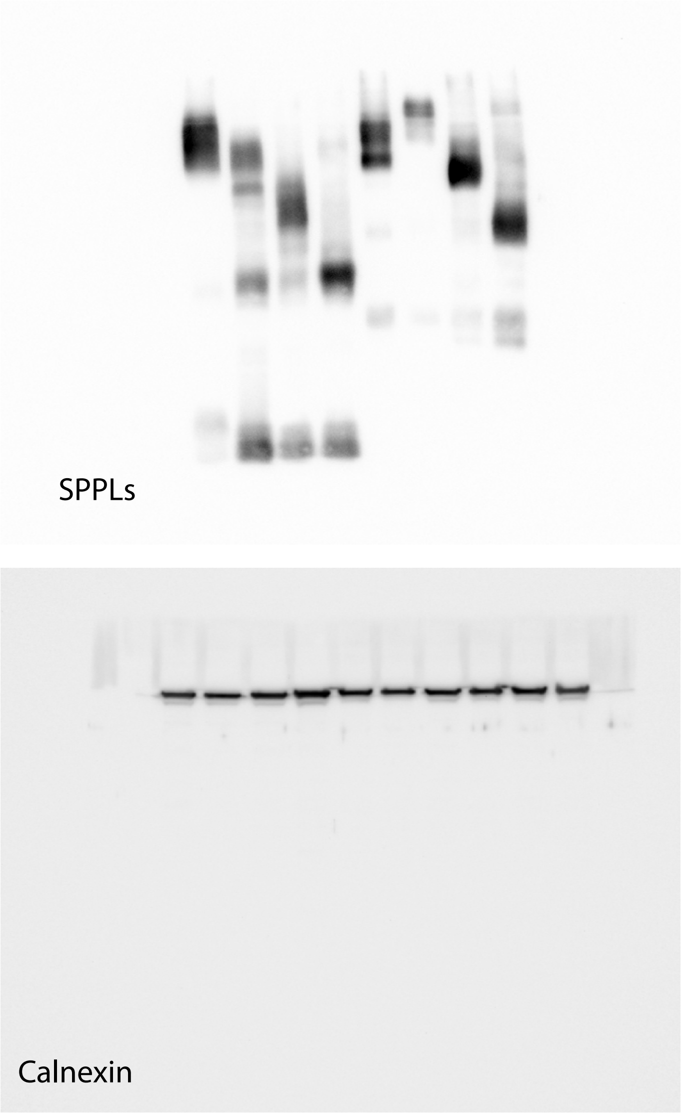

Figure 4a

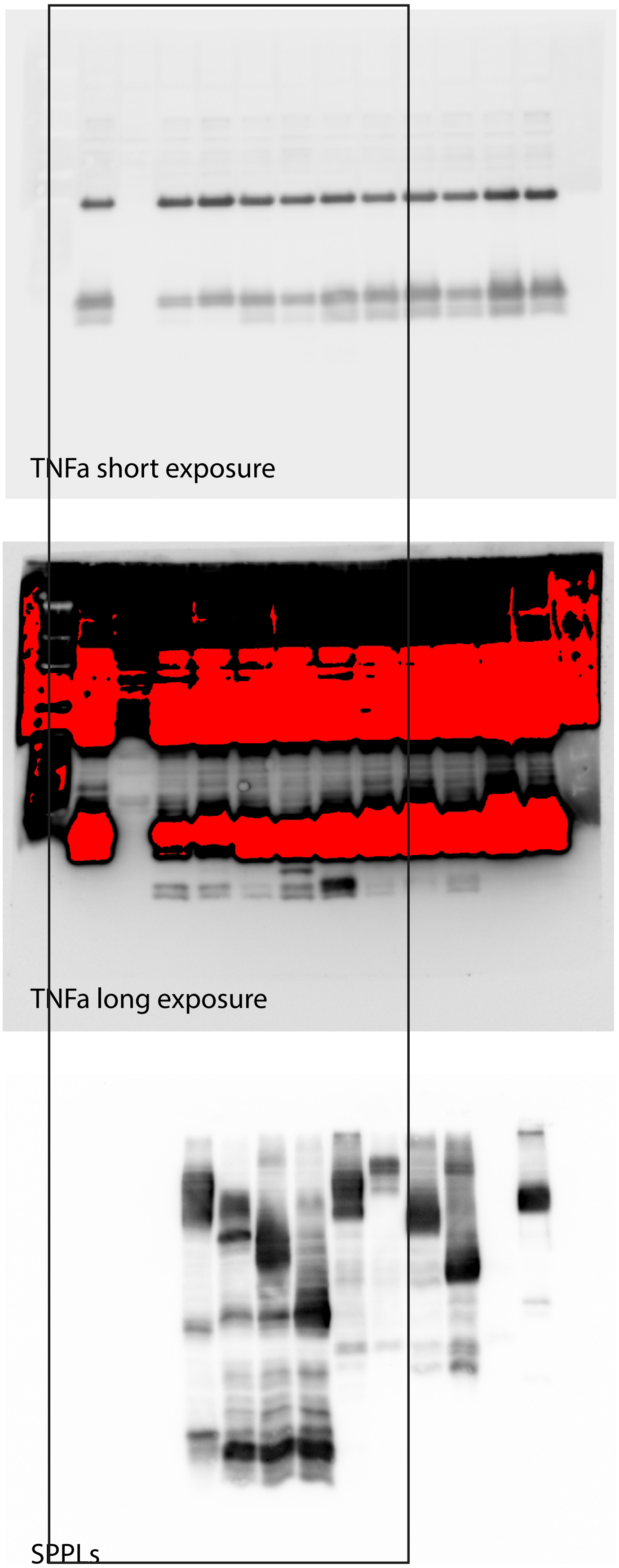

Figure 5a

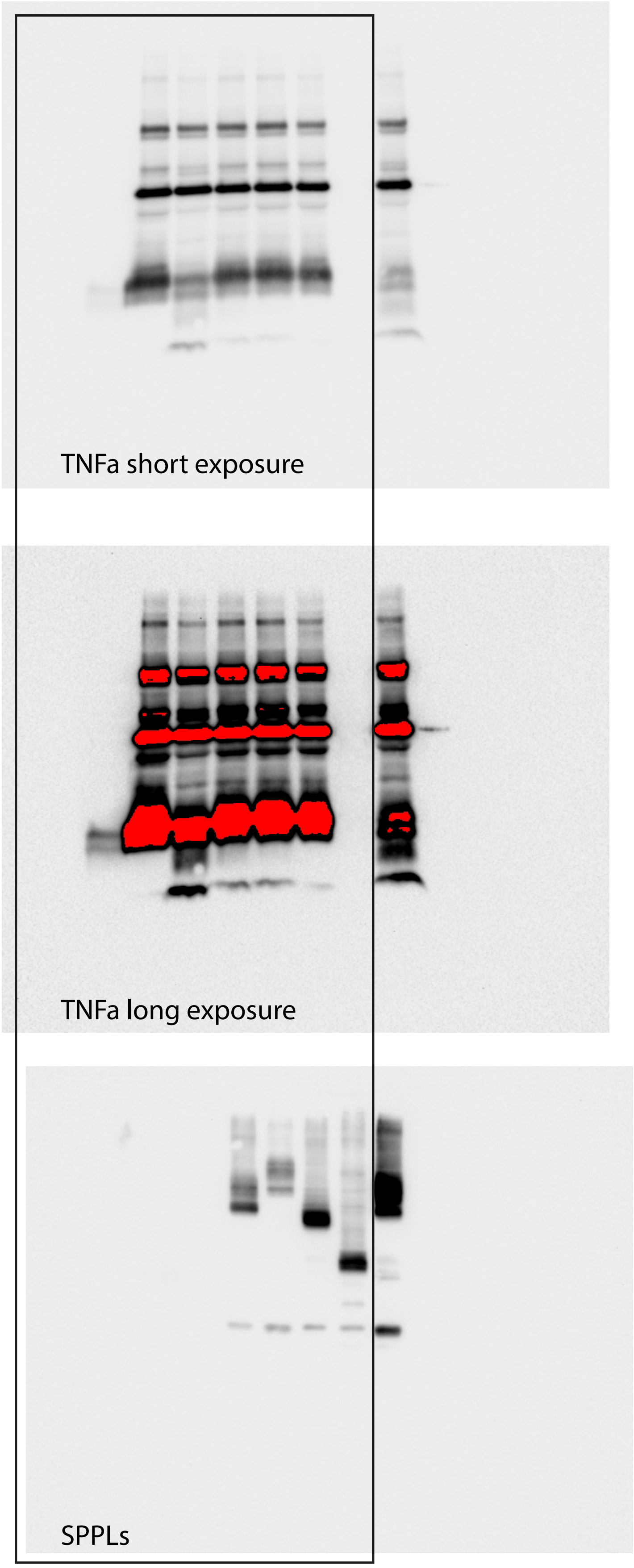

Figure 6a

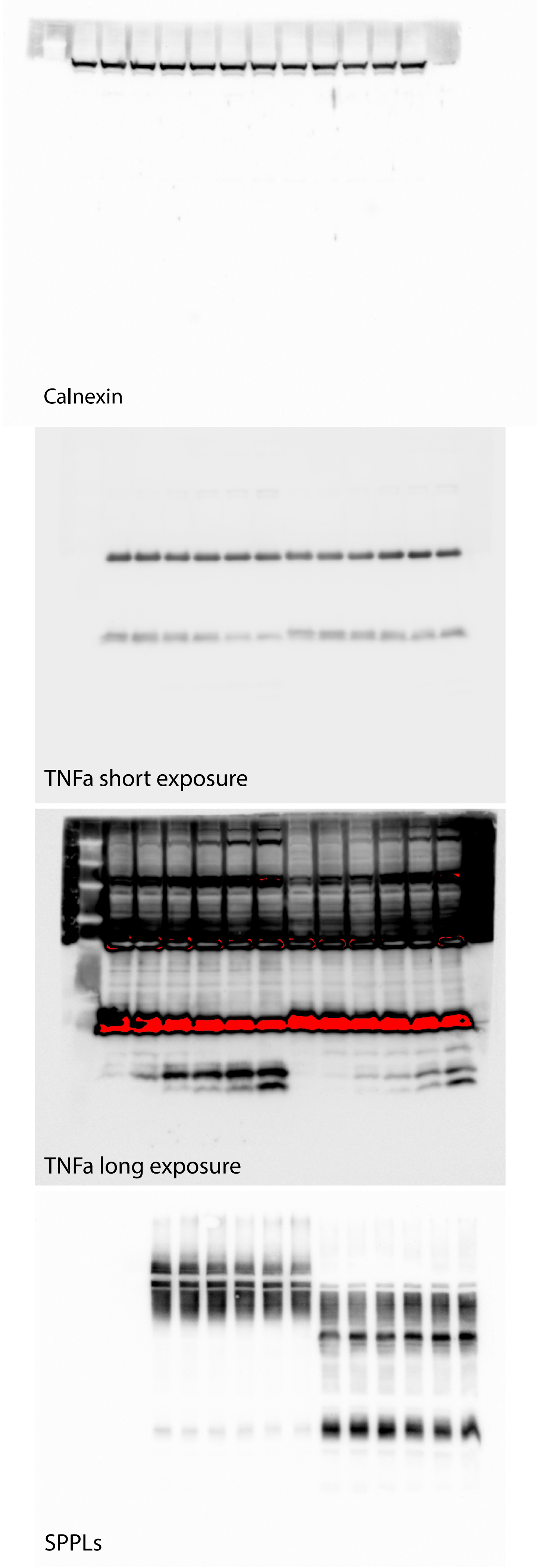

Figure 6b

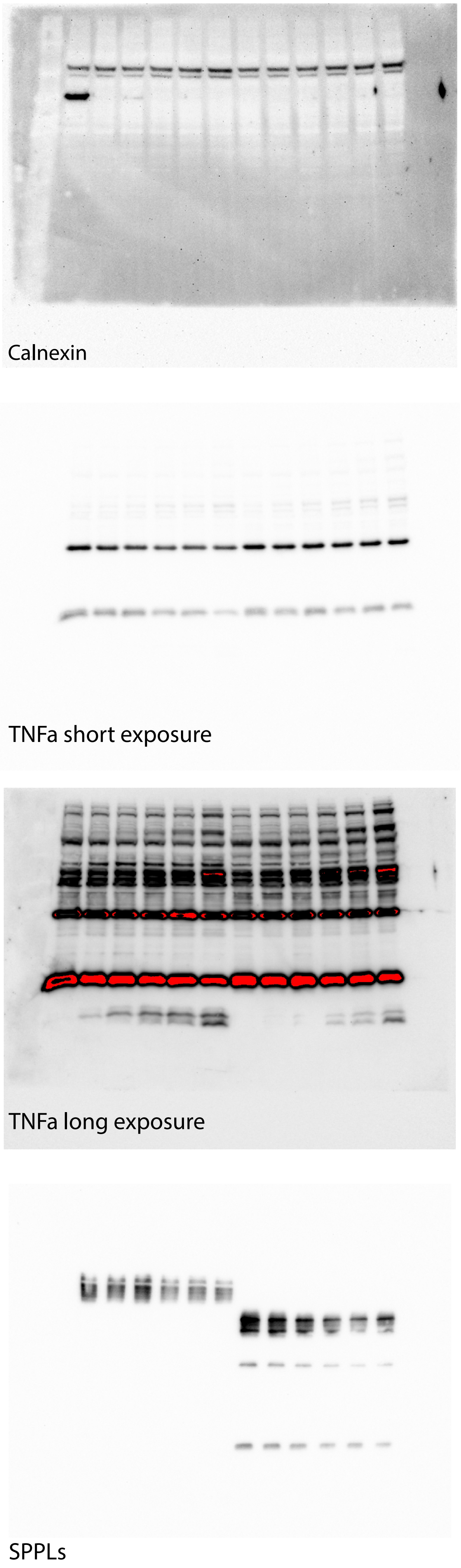

Figure 6c

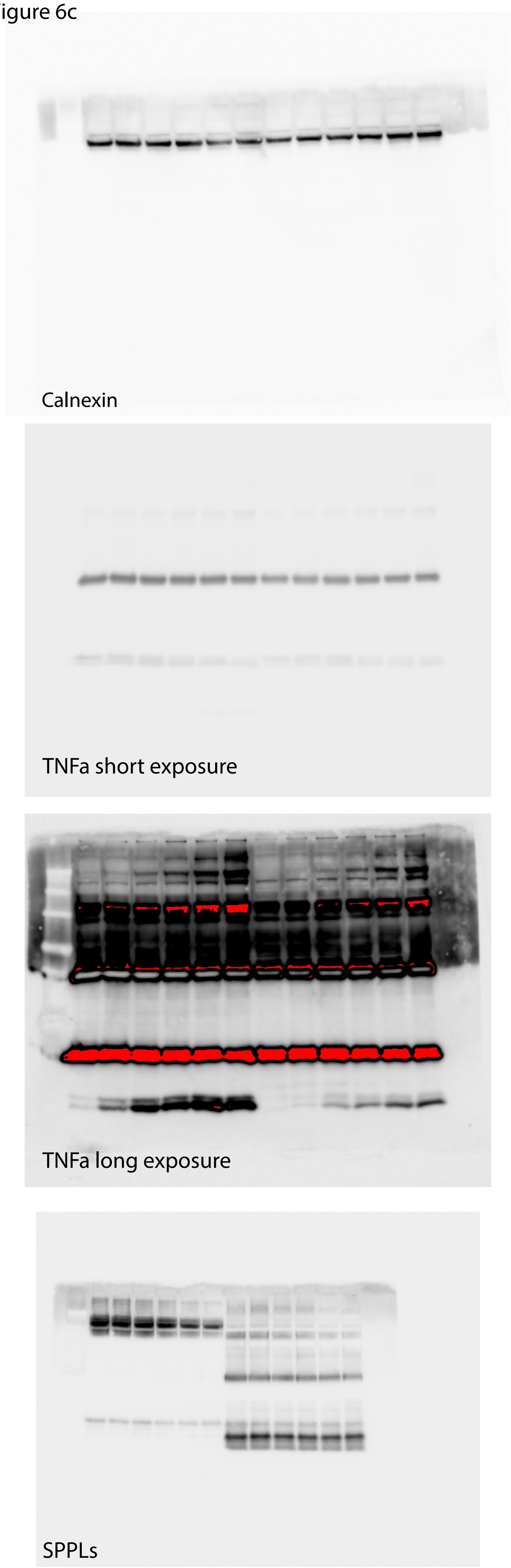

Figure 7a

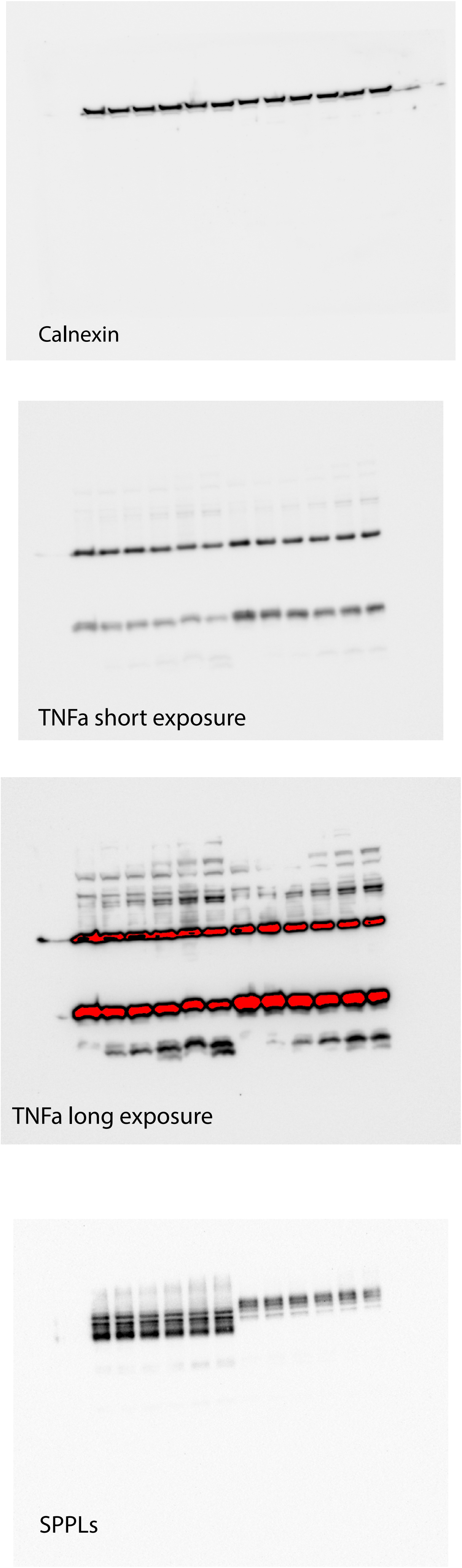

Figure 7b

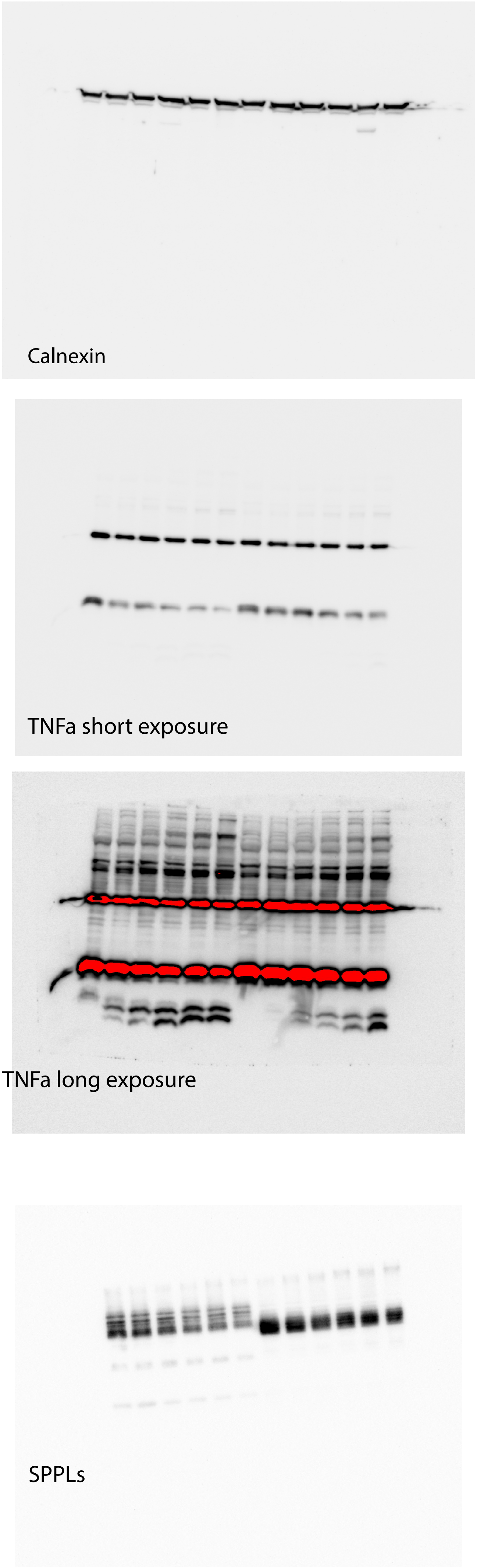

Figure 7c

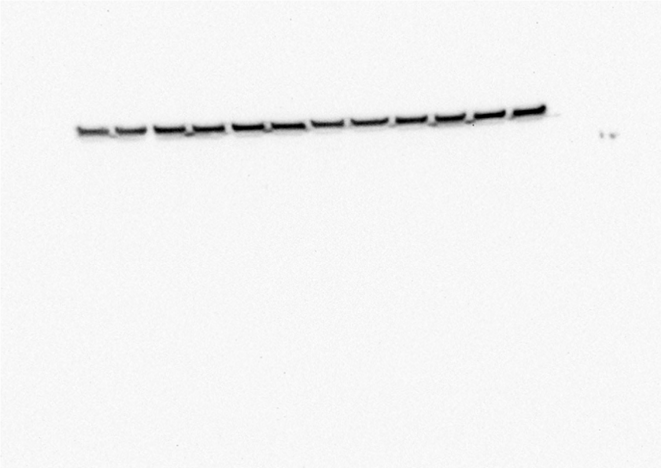

Calnexin

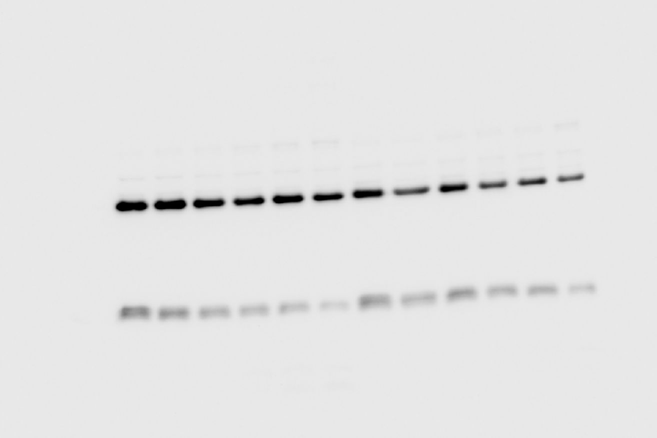

TNFa short exposure

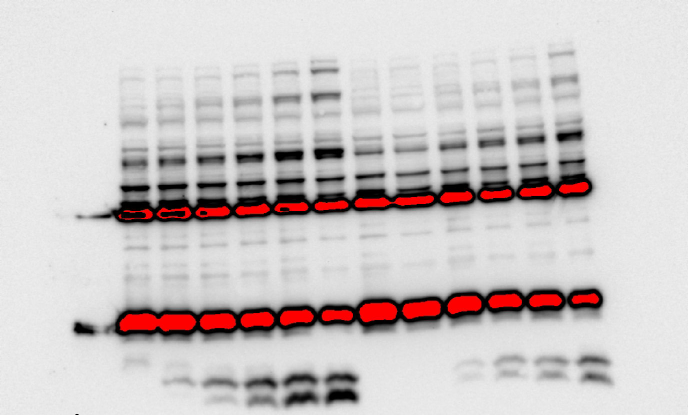

TNFa long exposure

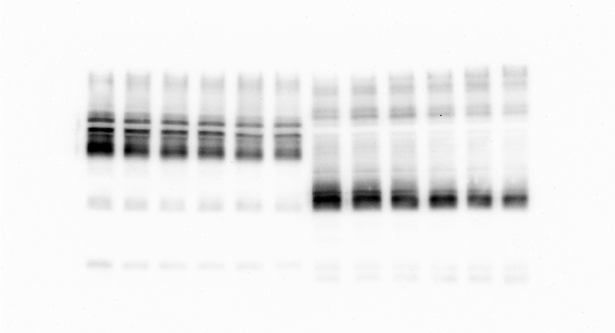

SPPLs

Figure 9a

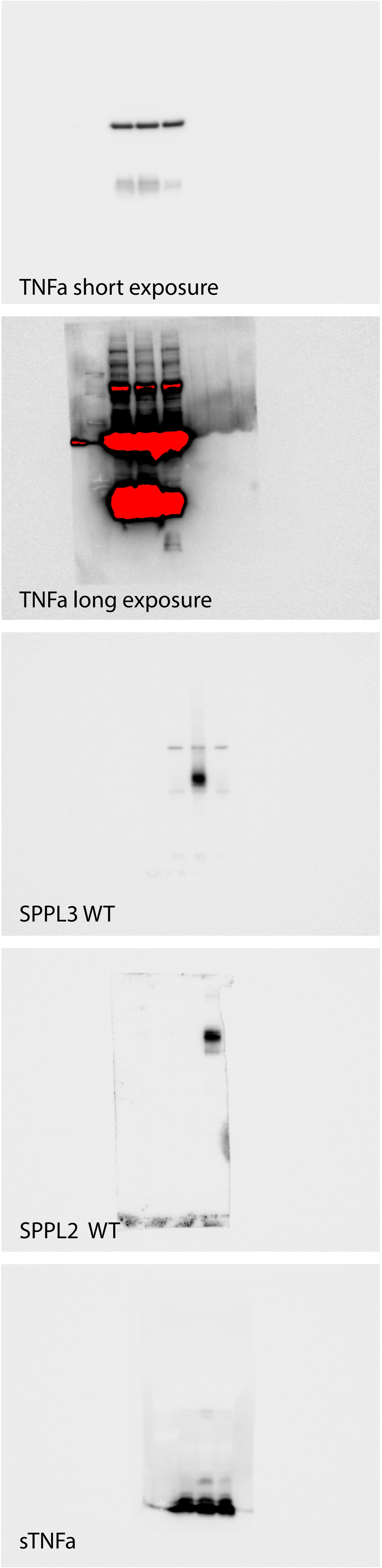

Figure 9d

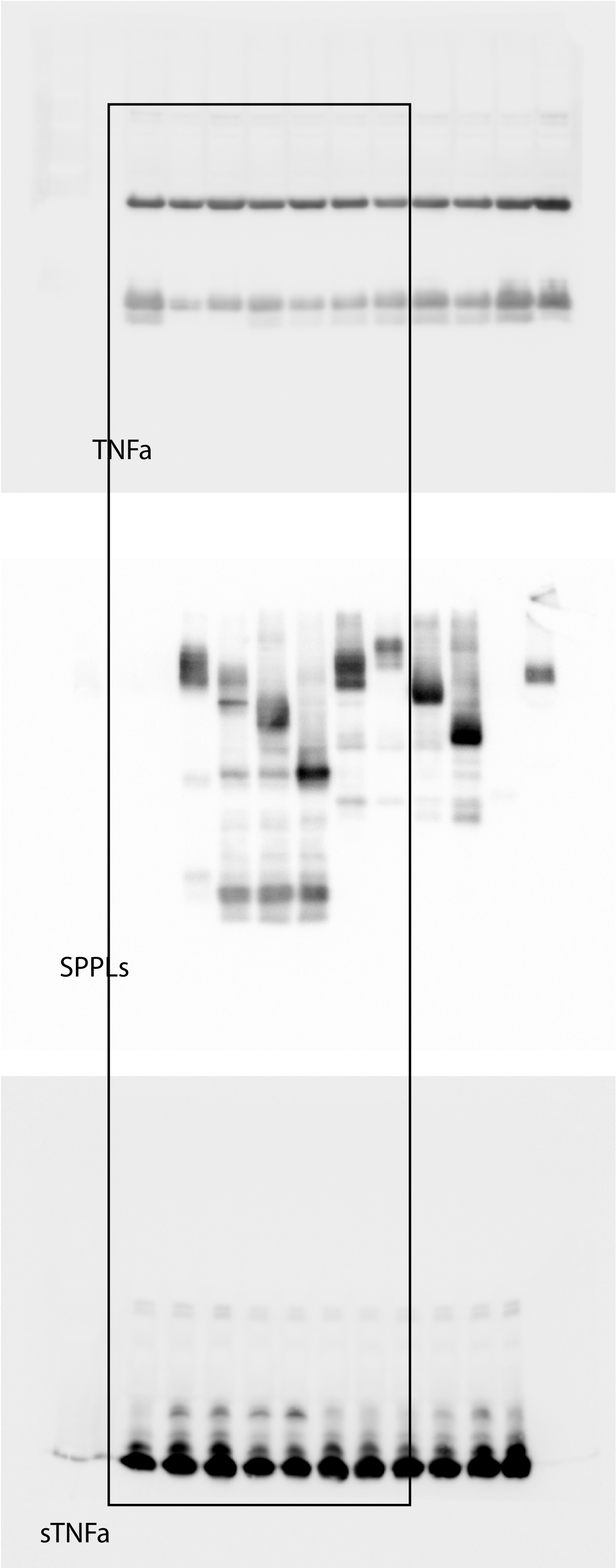

Figure 10a

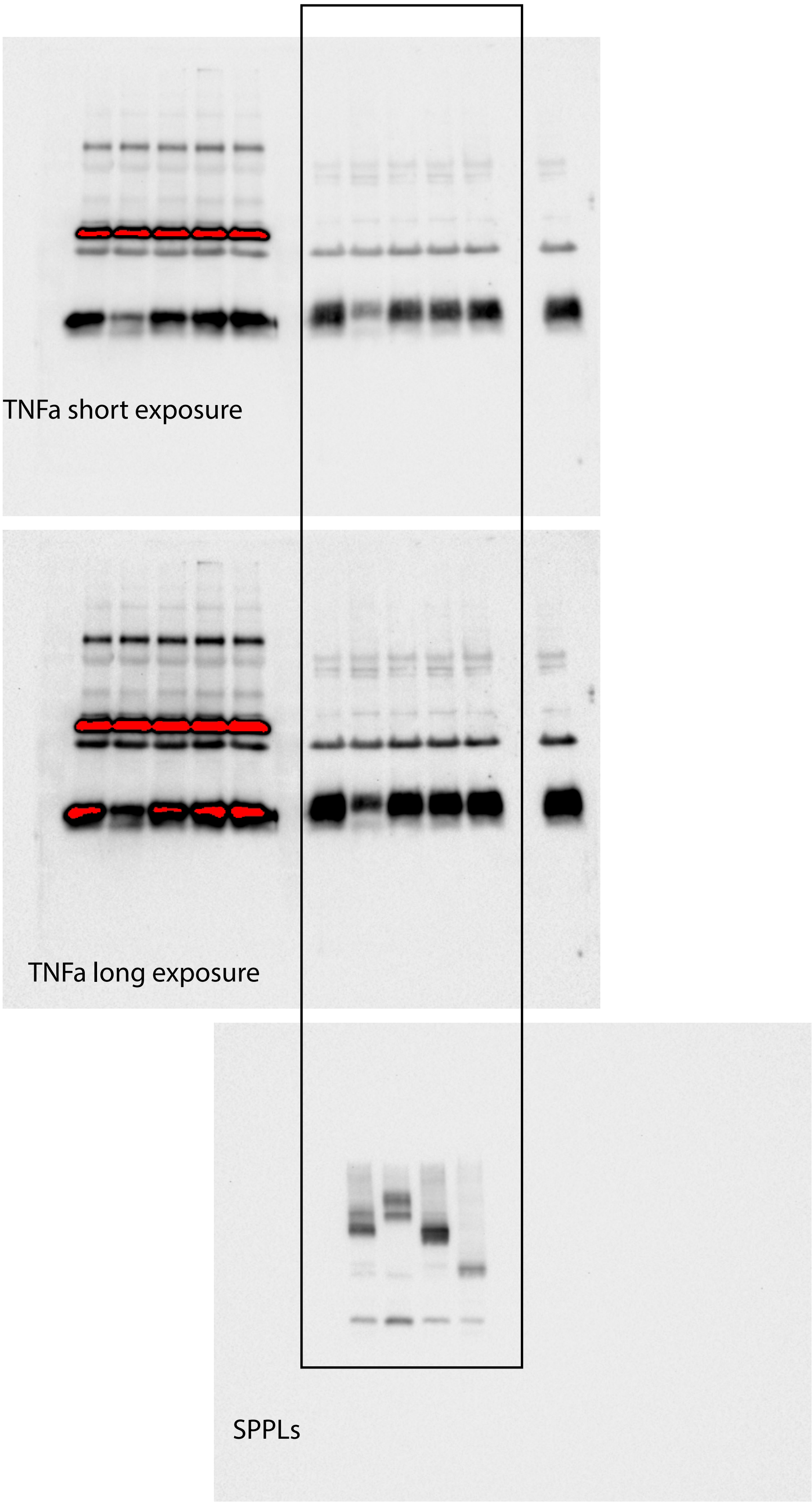

Supplement: Supplementary file 1 — Supplementary Information [file 42003_2025_8102_MOESM1_ESM.pdf]
