# Supplementary material for: The N-terminal PA domains of signal-peptide-peptidase-like 2 (SPPL2) proteases impact on TNFα cleavage
Source: Commun Biol. 2025 Apr 30;8:686. doi: 10.1038/s42003-025-08102-y (PMC12043953; doi:10.1038/s42003-025-08102-y)
Supplement: Supplementary file 2 — Description of Additional Supplementary Files [file 42003_2025_8102_MOESM2_ESM.pdf]

Description of Additional Supplementary Files

File name: Supplementary Data 1
Description: Numerical source data for all bar graphs shown in the main figures.
File name: Supplementary Data 2
Description: Raw data of the MALDI-TOF data in figure 8 and supplemental figure 6, as well as the sequence verifications of all chimeric enzymes
