# Supplementary material for: The N-terminal PA domains of signal-peptide-peptidase-like 2 (SPPL2) proteases impact on TNFα cleavage
Source: Commun Biol. 2025 Apr 30;8:686. doi: 10.1038/s42003-025-08102-y (PMC12043953; doi:10.1038/s42003-025-08102-y)
Supplement: Supplementary file 5 — Reporting summary [file 42003_2025_8102_MOESM5_ESM.pdf]

nature portfolio

Corresponding author(s): Sabine Hoeppner, Regina Fluhrer

Last updated by author(s): Apr 1, 2025

Reporting Summary

Nature Portfolio wishes to improve the reproducibility of the work that we publish. This form provides structure for consistency and transparency in reporting. For further information on Nature Portfolio policies, see our Editorial Policies and the Editorial Policy Checklist.

Statistics

For all statistical analyses, confirm that the following items are present in the figure legend, table legend, main text, or Methods section.

n/a	Confirmed	
☐	☒	The exact sample size (n) for each experimental group/condition, given as a discrete number and unit of measurement
☐	☒	A statement on whether measurements were taken from distinct samples or whether the same sample was measured repeatedly
☐	☒	The statistical test(s) used AND whether they are one- or two-sided<br>*Only common tests should be described solely by name; describe more complex techniques in the Methods section.*
☐	☒	A description of all covariates tested
☐	☒	A description of any assumptions or corrections, such as tests of normality and adjustment for multiple comparisons
☐	☒	A full description of the statistical parameters including central tendency (e.g. means) or other basic estimates (e.g. regression coefficient) AND variation (e.g. standard deviation) or associated estimates of uncertainty (e.g. confidence intervals)
☒	☐	For null hypothesis testing, the test statistic (e.g. F, t, r) with confidence intervals, effect sizes, degrees of freedom and P value noted<br>*Give P values as exact values whenever suitable.*
☒	☐	For Bayesian analysis, information on the choice of priors and Markov chain Monte Carlo settings
☒	☐	For hierarchical and complex designs, identification of the appropriate level for tests and full reporting of outcomes
☒	☐	Estimates of effect sizes (e.g. Cohen's d, Pearson's r), indicating how they were calculated

Our web collection on statistics for biologists contains articles on many of the points above.

Software and code

Policy information about availability of computer code

Data collection	Excel Version 2405
Data analysis	GraphPad Prism Version 9.4.1 (681)

For manuscripts utilizing custom algorithms or software that are central to the research but not yet described in published literature, software must be made available to editors and reviewers. We strongly encourage code deposition in a community repository (e.g. GitHub). See the Nature Portfolio guidelines for submitting code & software for further information.

Data

Policy information about availability of data

All manuscripts must include a data availability statement. This statement should provide the following information, where applicable:

- Accession codes, unique identifiers, or web links for publicly available datasets
- A description of any restrictions on data availability
- For clinical datasets or third party data, please ensure that the statement adheres to our policy

Resources availability
Further information and requests for resources and reagents should be directed to and will be fulfilled by Regina Fluhrer (regina.fluhrer@med.uni-augsburg.de).

Materials Availability

Cell lines and cDNA constructs generated in this study will be made available on request, but we may require a payment and/or a completed Materials Transfer Agreement if there is potential for commercial application.

Data Availability
No data with mandated deposition

Research involving human participants, their data, or biological material

Policy information about studies with human participants or human data. See also policy information about sex, gender (identity/presentation), and sexual orientation and race, ethnicity and racism.

Reporting on sex and gender	n.a.
Reporting on race, ethnicity, or other socially relevant groupings	n.a.
Population characteristics	n.a.
Recruitment	n.a.
Ethics oversight	n.a.

Note that full information on the approval of the study protocol must also be provided in the manuscript.

Field-specific reporting

Please select the one below that is the best fit for your research. If you are not sure, read the appropriate sections before making your selection.

☒ Life sciences ☐ Behavioural & social sciences ☐ Ecological, evolutionary & environmental sciences

For a reference copy of the document with all sections, see nature.com/documents/nr-reporting-summary-flat.pdf

Life sciences study design

All studies must disclose on these points even when the disclosure is negative.

Sample size	This is a exploratory early-stage study, aiming to identify promising trends or effects rather than achieving broad generalizability. A sample size of 3–7 can provide preliminary evidence of an effect, while larger sample sizes improve statistical power. In this study smaller sample sizes was sufficient due to the used controlled model systems (cell lines and in vitro assay), compared to the biological variability of clinical studies with human subjects.
Data exclusions	Many results are based on the densitometric quantification of Western blots. If the transfer was not successful, the entire experiment was excluded.
Replication	All experiments were reproducible when loading variations and expression differences were accounted for in the quantification and statistical analysis
Randomization	N-terminal chimeras were always grouped with the protease body containing the wild type to compare the effect of the N-terminus independently of the protease body.
Blinding	Investigators were not blinded to group allocation during data collection and analysis because the focus was on generating hypotheses rather than confirmatory findings. Additionally, findings from one part of the study guided the next steps and the design of additional experiments

Reporting for specific materials, systems and methods

We require information from authors about some types of materials, experimental systems and methods used in many studies. Here, indicate whether each material, system or method listed is relevant to your study. If you are not sure if a list item applies to your research, read the appropriate section before selecting a response.

Materials & experimental systems

n/a	Involved in the study
☐	☒ Antibodies
☐	☒ Eukaryotic cell lines
☒	☐ Palaeontology and archaeology
☒	☐ Animals and other organisms
☒	☐ Clinical data
☒	☐ Dual use research of concern
☒	☐ Plants

Methods

n/a	Involved in the study
☒	☐ ChIP-seq
☒	☐ Flow cytometry
☒	☐ MRI-based neuroimaging

Antibodies

Antibodies used

anti-HA-Fluorescein (Roche, 11988506001, clone 3F10); anti-Lamp1 (cell signalling, #9091, clone D2D11); anti-GRP78/BIP (Abcam, ab21685, pAb); anti-GM130 (BD Bioscience, 610823, clone 35); anti-FLAG (Sigma, F7425, pAB); anti-FLAG beads (Sigma, A2220, clone M2); anti-FLAG (Merck, F1804, clone M2); anti-V5 (Invitrogen, R960-25, clone SV5-Pk1); anti-Calnexin (Enzo Life Sciences, ADI-SPA-860-F, clone pAB); anti-HA HRP (Roche, 11867423001, clone 3F10); anti-mouse HRP (Promega, W4021, pAB); anti-rabbit HRP (Promega, W4011, pAB); anti-rat HRP (Merck, AP136P, pAB); goat anti-rabbit, Alexa Fluor™ 555 (Thermo Fisher, A-21428, pAB); goat anti-mouse, Alexa Fluor™ 555 (Thermo Fisher, A32727, pAB); anti-SPPL3 (Regina Feederle, 7F9); anti-SPPL2b (Regina Feederle, 2G8)

Validation

anti-HA-Fluorescein (Chan CH, Lin P, Yang TY, Bao BY, Jhong JY, Weng YP, Lee TH, Cheng HF, Lu TL. Epithelial polarization in the 3D matrix requires MST3 signaling to regulate ZO-1 position. PLoS One. 2023 May 8;18(5):e0285217. doi: 10.1371/journal.pone.0285217. PMID: 37155619; PMCID: PMC10166550.); anti-Lamp1 (Zhao M, Zhang S, Wan W, Zhou C, Li N, Cheng R, Yu Y, Ouyang X, Zhou D, Jiao J, Xiong X. Coxiella burnetii effector CvpE maintains biogenesis of Coxiella-containing vacuoles by suppressing lysosome tubulation through binding PI(3)P and perturbing PIKfyve activity on lysosomes. Virulence. 2024 Dec;15(1):2350893. doi: 10.1080/21505594.2024.2350893. Epub 2024 May 9. PMID: 38725096; PMCID: PMC11085968.), anti-GRP78/BIP (Datta P, Allamargot C, Hudson JS, Andersen EK, Bhattarai S, Drack AV, Sheffield VC, Seo S. Accumulation of non-outer segment proteins in the outer segment underlies photoreceptor degeneration in Bardet-Biedl syndrome. Proc Natl Acad Sci U S A. 2015 Aug 11;112(32):E4400-9. doi: 10.1073/pnas.1510111112. Epub 2015 Jul 27. PMID: 26216965; PMCID: PMC4538681.), anti-GM130 (Torii T, Shirai R, Kiminami R, Nishino S, Sato T, Sawaguchi S, Fukushima N, Seki Y, Miyamoto Y, Yamauchi J. Hypomyelinating Leukodystrophy 10 (HLD10)-Associated Mutations of PYCR2 Form Large Size Mitochondria, Inhibiting Oligodendroglial Cell Morphological Differentiation. Neurol Int. 2022 Dec 16;14(4):1062-1080. doi: 10.3390/neurolint14040085. PMID: 36548190; PMCID: PMC9787162.); anti-FLAG pAB (Peinado JR, Chaplot K, Jarvela TS, Barbieri EM, Shorter J, Lindberg I. Sequestration of TDP-43216-414 Aggregates by Cytoplasmic Expression of the proSAAS Chaperone. ACS Chem Neurosci. 2022 Jun 1;13(11):1651-1665. doi: 10.1021/acschemneuro.2c00156. Epub 2022 May 12. PMID: 35549000; PMCID: PMC9731516.), anti-FLAG M2 beads (Liu H, Mei FC, Yang W, Wang H, Wong E, Cai J, Toth E, Luo P, Li YM, Zhang W, Cheng X. Epac1 inhibition ameliorates pathological angiogenesis through coordinated activation of Notch and suppression of VEGF signaling. Sci Adv. 2020 Jan 1;6(1):eaay3566. doi: 10.1126/sciadv.aay3566. PMID: 31911948; PMCID: PMC6938696.), anti-FLAG M2 (Srivastava M, Duan G, Kershaw NJ, Athanasopoulos V, Yeo JH, Ose T, Hu D, Brown SH, Jergic S, Patel HR, Pratama A, Richards S, Verma A, Jones EY, Heissmeyer V, Preiss T, Dixon NE, Chong MM, Babon JJ, Vinuesa CG. Roquin binds microRNA-146a and Argonaute2 to regulate microRNA homeostasis. Nat Commun. 2015 Feb 20;6:6253. doi: 10.1038/ncomms7253. PMID: 25697406; PMCID: PMC4346627.); anti-V5 (Gouveia Roque C, Chung KM, McCurdy EP, Jagannathan R, Randolph LK, Herline-Killian K, Baleriola J, Hengst U. CREB3L2-ATF4 heterodimerization defines a transcriptional hub of Alzheimer's disease gene expression linked to neuropathology. Sci Adv. 2023 Mar 3;9(9):eadd2671. doi: 10.1126/sciadv.add2671. Epub 2023 Mar 3. PMID: 36867706; PMCID: PMC9984184.), anti-Calnexin (Werner NT, Högel P, Güner G, Stelzer W, Wozny M, Aßfalg M, Lichtenthaler SF, Steiner H, Langosch D. Cooperation of N- and C-terminal substrate transmembrane domain segments in intramembrane proteolysis by γ-secretase. Commun Biol. 2023 Feb 15;6(1):177. doi: 10.1038/s42003-023-04470-5. PMID: 36792683; PMCID: PMC9931712.), anti-HA HRP (Kroening S, Neubauer E, Wessel J, Wiesener M, Goppelt-Struebe M. Hypoxia interferes with connective tissue growth factor (CTGF) gene expression in human proximal tubular cell lines. Nephrol Dial Transplant. 2009 Nov;24(11):3319-25. doi: 10.1093/ndt/gfp305. Epub 2009 Jun 23. PMID: 19549692.), anti-mouse HRP (Hu D, Zhou Z, Davidson NE, Huang Y, Wan Y. Novel insight into KLF4 proteolytic regulation in estrogen receptor signaling and breast carcinogenesis. J Biol Chem. 2012 Apr 20;287(17):13584-97. doi: 10.1074/jbc.M112.343566. Epub 2012 Mar 2. PMID: 22389506; PMCID: PMC3340146.), anti-rabbit HRP (Hesse E, Saito H, Kiviranta R, Correa D, Yamana K, Neff L, Toben D, Duda G, Atfi A, Geoffroy V, Horne WC, Baron R. Zfp521 controls bone mass by HDAC3-dependent attenuation of Runx2 activity. J Cell Biol. 2010 Dec 27;191(7):1271-83. doi: 10.1083/jcb.201009107. Epub 2010 Dec 20. PMID: 21173110; PMCID: PMC3010073.), goat anti-rat HRP (Chen IT, Hsu PH, Hsu WC, Chen NJ, Tseng PH. Polyubiquitination of Transforming Growth Factor β-activated Kinase 1 (TAK1) at Lysine 562 Residue Regulates TLR4-mediated JNK and p38 MAPK Activation. Sci Rep. 2015 Jul 20;5:12300. doi: 10.1038/srep12300. PMID: 26189595; PMCID: PMC4507259.), Goat anti-Rabbit, Alexa Fluor™ 555 (Daeschler SC, So KJW, Feinberg K, Manoraj M, Cheung J, Zhang J, Mirmoeini K, Santerre JP, Gordon T, Borschel GH. A functional tacrolimus-releasing nerve wrap for enhancing nerve regeneration following surgical nerve repair. Neural Regen Res. 2025 Jan 1;20(1):291-304. doi: 10.4103/NRR.NRR-D-22-01198. Epub 2024 Jan 31. PMID: 38767493; PMCID: PMC11246136.), Goat anti-Mouse, Alexa Fluor™ 555 (Sun JX, Yao Y, Li WX, Su X, Yang H, Lu Z, Liu C, Xu XH, Jin L. Upregulation of GPR133 expression impaired the phagocytosis of macrophages in recurrent spontaneous miscarriage. Epigenetics. 2024 Dec;19(1):2337087. doi: 10.1080/15592294.2024.2337087. Epub 2024 Apr 2. PMID: 38564758; PMCID: PMC10989699.); anti-SPPL3 (Voss M, Fukumori A, Kuhn PH, Künzel U, Klier B, Grammer G, Haug-Kröper M, Kremmer E, Lichtenthaler SF, Steiner H, Schröder B, Haass C, Fluhrer R. Foamy virus envelope protein is a substrate for signal peptide peptidase-like 3 (SPPL3). J Biol Chem. 2012 Dec 21;287(52):43401-9. doi: 10.1074/jbc.M112.371369. Epub 2012 Nov 6. PMID: 23132852; PMCID: PMC3527927.); anti-SPPL2b (Martin L, Fluhrer R, Reiss K, Kremmer E, Saftig P, Haass C. Regulated intramembrane proteolysis of Bri2 (Itm2b) by ADAM10 and SPPL2a/SPPL2b. J Biol Chem. 2008 Jan 18;283(3):1644-1652. doi: 10.1074/jbc.M706661200. Epub 2007 Oct 25. PMID: 17965014)

Eukaryotic cell lines

Policy information about cell lines and Sex and Gender in Research

Cell line source(s)	TR SPPL2a/b double knockout cells based on the T-REx™-293 Cell Line System (Spitz C, Schlosser C, Guschtschin-Schmidt N, Stelzer W, Menig S, Götz A, Haug-Kröper M, Scharnagl C, Langosch D, Muhle-Goll C, Fluhrer R. Non-canonical Shedding of TNFα by SPPL2a Is Determined by the Conformational Flexibility of Its Transmembrane Helix. iScience. 2020 Nov 5;23(12):101775. doi: 10.1016/j.isci.2020.101775. PMID: 33294784; PMCID: PMC7689174.)
Authentication	DNA sequence verification was performed through Sanger sequencing, before cell transfection
Mycoplasma contamination	All cell lines were tested negative for mycoplasma contamination
Commonly misidentified lines (See ICLAC register)	n.a.

Plants

Seed stocks	n.a.
Novel plant genotypes	n.a.
Authentication	n.a.
